# Mechanism of self-recovery of hydrophobicity after surface damage of lotus leaf

**DOI:** 10.1186/s13007-024-01174-7

**Published:** 2024-03-21

**Authors:** Li Wang, Lichun Shu, Qin Hu, Xingliang Jiang, Hang Yang, Huan Wang, Lipeng Rao

**Affiliations:** Xuefeng Mountain Energy Equipment Safety National Observation and Research Station of Chongqing University, Chongqing, 400044 China

**Keywords:** Hydrophobic, Self-recovery, Lotus leaf, Surface damage, Graded roughness

## Abstract

The surfaces of lotus leaves with micro- and nano-waxy cuticle structures are superhydrophobic and possess a self-healing ability to regain hydrophobicity after damage. Inspired by this phenomenon, the problem of water-repellent coatings used in natural environments failing to perform after damage can be solved if these coatings are endowed with rapid self-repair and self-growth functions. However, there has been almost no exploration into the hydrophobicity self-repair process in lotus leaves. The changes in surface morphology during the hydrophobicity recovery process are not understood. There is a lack of research on the hydrophobicity recovery in lotus leaves. In this study, the damage and recovery experiments on lotus leaf surfaces were carried out in an artificial climate chamber, and the water repellency recovery process and typical water repellency roughness parameters regained time were obtained. Upon analyzing the differences in the recovery process of different damage types, the recovery mechanism after lotus leaf surface damage was obtained. Finally, it was found that the microscopic roughness determined the static contact angle (WCA) of the lotus leaf surface, and the nanoscopic roughness determined the rolling angle (SA). The dual factors of the recovery of the extruded epidermal tissue and the regeneration of the epidermal wax crystals determined the hydrophobicity recovery process in damaged lotus leaves.

## Introduction

After billions of years of evolution, nature has created natural superhydrophobic surfaces with special wettability represented by lotus leaves, hogwash, water striders, etc. [1]. Take the "lotus leaf effect" as an example; water droplets maintain a complete droplet form on the lotus leaf surface. They can roll naturally from the lotus leaf surface because of wind and other external disturbances [2]. Inspired by this phenomenon, researchers have begun to replicate similar functional surfaces for use in all corners of social production and life [3–5].

Ice cover can substantially damage various infrastructure elements such as transmission lines, towers, wind turbines, and other equipment [6–8]. This damage, in turn, has the potential to compromise equipment and facility efficiency, posing significant safety hazards. Due to the lack of energy supply and active triggering, passive anti-icing methods represented by superhydrophobic surfaces have received increasing attention as an important method to address surface ice cover [9]. Researchers have prepared a variety of superhydrophobic surfaces with low surface energies and micro-nanostructures [10, 11]. However, the poor durability of artificial surfaces that are overly dependent on surface morphology and chemical composition affects their practical applications [12]. In addition, the surface energy of superhydrophobic surfaces that adsorb dust and dirt from the environment becomes high, leading to a failure of superhydrophobic properties [13]. Therefore, a robust and durable superhydrophobic surface is an important research direction for the wider and long-lasting use of superhydrophobic materials [14]. However, to prepare solid and durable superhydrophobic surfaces, methods to increase the adhesion between the superhydrophobic surface and the substrate material have been adopted. There is a drawback to this method. If the material surface is damaged, the superhydrophobic surface fails.

To investigate the wettability recovery mechanism in natural superhydrophobic surfaces, researchers observed the surface wettability recovery process in surviving and dead clover leaves using plasma-treated clover leaves [15]. In addition, the wetting properties of kale’s waxy surface were studied, and the recovery time curve of the waxy surface after mechanical damage was analyzed [16].

The published research on wax recovery in plant surfaces does not explicitly study the damage and recovery process of surface structures and the effect of different damage types on plant surface wettability. The self-healing processes of lotus superhydrophobic properties and surface roughness were explored to provide a reference for the design of self-healing superhydrophobic surfaces. In this study, we mainly studied changes in the surface wettability of lotus leaves after physical, chemical, and icing-deicing cycle damage and its recovery process over time.

## Experimental section

### Materials

**Fig. 1:**
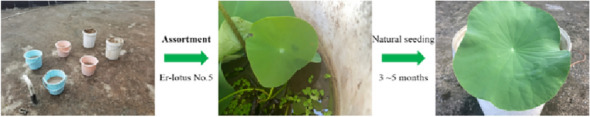
The process of growing lotus leaves

The lotus leaves used in the experiment were common ornamental lotus leaves. To study the recovery process in lotus leaves, it is necessary to use the surviving lotus leaves for experiments. Therefore, the leaves must not be removed from the plant for experiments. The sprouted lotus root seeds were buried in a bucket of lotus pond mud. The growth process does not control environmental parameters (e.g., pH, temperature, etc.) to obtain lotus leaf plants under natural growth (Fig. 1).

### Characterization techniques

(1) *Water repellency measurement*: The measurement of the hydrostatic contact angle (WCA) was conducted using an SDC-100 optical contact angle meter. Since the hydrophobicity degree varies from one part of the lotus leaf surface to another, three different surface locations were randomly measured and averaged. The laboratory temperature was maintained at a constant 25 °C during the measurements. After the damage was applied, slices were made at intervals on the lotus leaf samples, and the wettability test was carried out on these slices. In addition, the testing intervals were gradually increased.

(2) *Surface roughness measurement*: At least three roughness measurements were made on each sample using a LEXT OLS4000 3D confocal microscope. To minimize the influence of surface waxes by the dispersion of different parts of the leaf surface and the time of sample preparation, the measurement position was chosen as the stemless position on the lotus leaf surface. Immediately after slicing, roughness measurements were taken. Surface arithmetic mean height (Sa) and surface root-mean-square height (Sq) were selected as area surface roughness parameters according to ISO 25178. This parameter was used to analyze the correlation between surface roughness and surface wetting capacity.

(3) *Surface wax growth process measurements*: The surface wax condition was obtained using transmission scanning electron microscopy. To avoid melting the surface waxes during drying, the lotus leaf surface slices were dried at a critical temperature. The dried lotus leaf slices were first coated with gold spraying, and then the surface morphology was tested.

### Damage types

To investigate the wettability and surface roughness recovery of leaves after damage, the surfaces of the leaves were treated using three methods, namely, physical and chemical damage and the "ice-covering–deicing" cycle.

(1) *Physical damage*

To study the effect of different degrees of physical damage on the lotus leaf surface, two damage methods, minor and severe, were used. During physical damage, damage and reference groups were obtained by damaging only part of the lotus leaf surface.

**Fig. 2:**
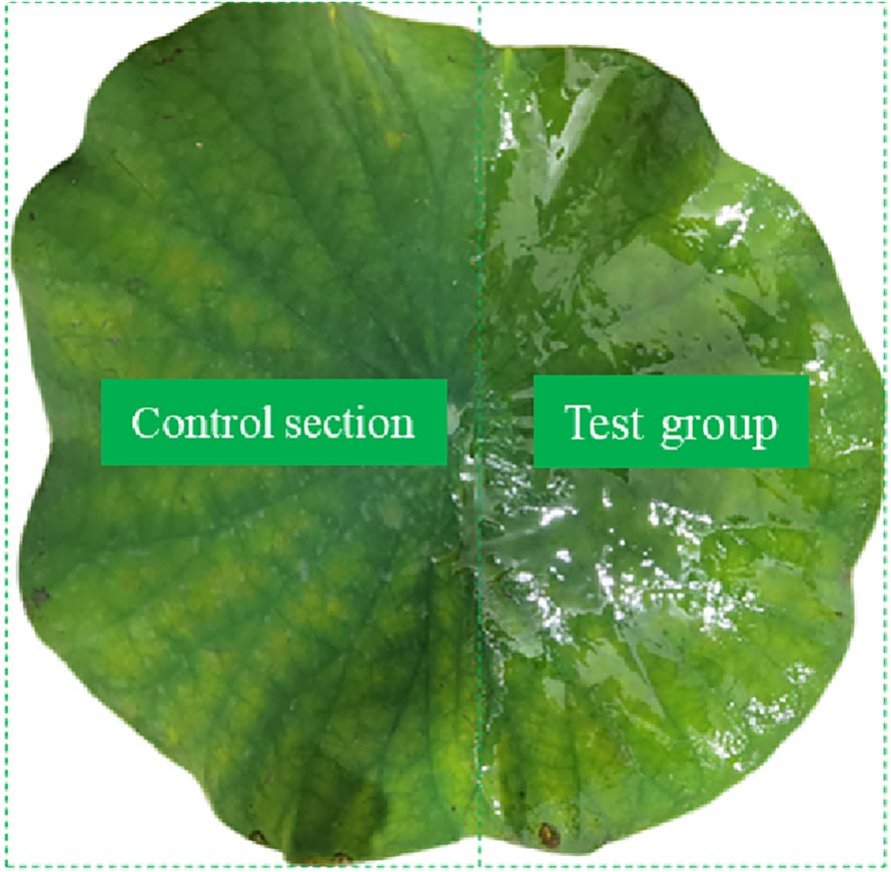
Two-component glue is applied to the surface of lotus leaf

The principle of minor physical damage is to damage only the wax layer on the lotus leaf surface as much as possible. Researchers usually use a two-component epoxy adhesive (3 M DP100 PLUS Fast Cure Epoxy Adhesive) to peel the wax layer from the epidermal plant cells. The two-component fast-curing epoxy adhesive was provided by 3 M, model 3 M DP100 PLUS. The mixed two-component epoxy resin adhesive was spread evenly on the lotus leaf surface, as shown in Fig. 2. The curing time of the room epoxy adhesive determines the degree of wax removal during peeling. In this study, peeling after curing at room temperature for 24 h (full cure time) was used to discuss minor physical damage [17, 18].

**Fig. 3:**
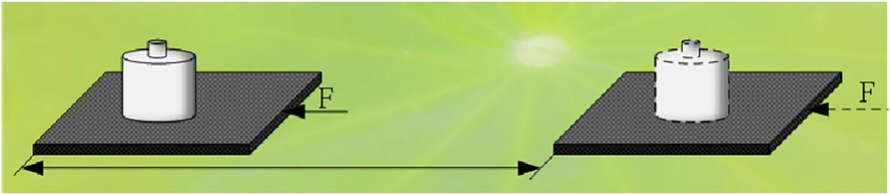
Severe physical damage method

The severe physical damage [19] method was designed by the abrasion resistance test standard ASTM: D 968-93. First, the upper lotus leaf surface was fixed on a horizontal surface, 1500 grit sandpaper with a 500 g load was placed on the lotus leaf surface, and the sandpaper was dragged to inflict severe physical damage to the lotus leaf surface, as shown in Fig. 3. A 1500 mesh sandpaper and 500-g weight were purchased from a local market. Considering the fragility of the lotus leaf surface, it was worn only 10 times in this experiment.

(2) *Chemical damage*

Chloroform completely dissolves the wax layer in the plant epidermis. The common method is to remove the whole lotus leaf after submerging it in a chloroform solution for 30 s, and then a lotus leaf surface is obtained after chemical damage [20] is obtained after the chloroform evaporates. Chloroform s Since this study was conducted on whole lotus leaves, there were operational difficulties in submerging the leaves in chloroform. Therefore, a cotton swab dipped in chloroform was used to treat the lotus leaf surfaces to obtain a chemically damaged surface.

(3) *"Ice-covering-deicing" cycle damage*

**Fig. 4:**
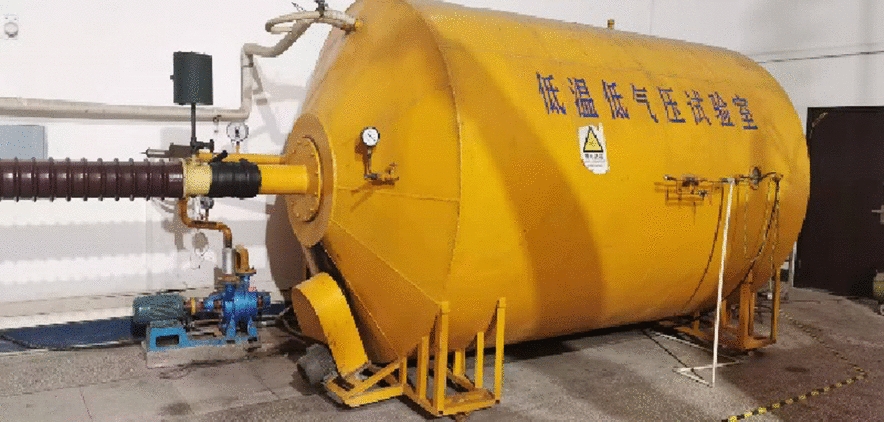
Low temperature and low air pressure test chamber

A low-temperature, low-pressure climate chamber simulates an overlying ice environment. It is 3.8 m long, with an inner diameter of 2 m and a minimum temperature of − 45 °C, as shown in Fig. 4. The climate chamber utilizes a nozzle recommended by the International Electrotechnical Commission (IEC). It produces water droplets with diameters from 10 to 100 μm. The water flow is controlled using the IEEE 1783TM-2009 standard and tested for icing.

**Fig. 5:**
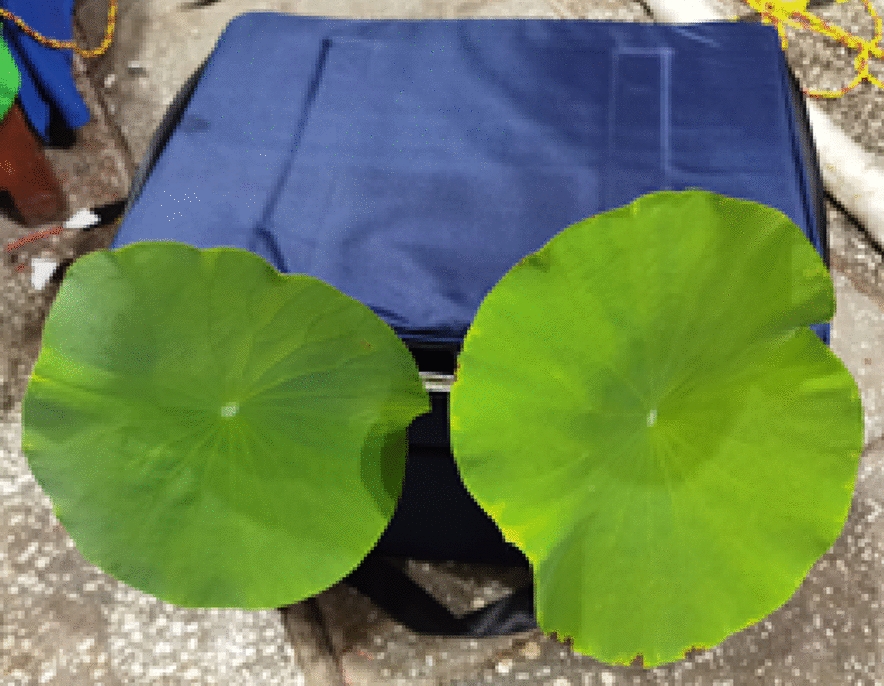
Lotus leaf plant insulation layout

The ambient temperature was set to − 5 °C, the ambient humidity was about 90%, the wind speed was controlled at 2 m/s, the water conductivity was 370 μS/cm, and the water droplet diameters were 80–100 μm. Since lotus leaves have a poor low-temperature tolerance, the lotus plant roots were placed in a holding box, as shown in Fig. 5. Whole lotus leaves and the holding box as a whole were placed in an artificial climate chamber, and only the lotus leaf surface was covered with ice.

The overall process of the "ice-covering–deicing" cycle occurred as follows: after 10 min of over-icing, the leaves were removed from the chamber and placed at room temperature (25 °C) for natural deicing. When the surface ice was completely removed, a single "ice-covering–deicing" cycle was completed. To discuss the effect of the number of "ice-covering–deicing" cycles on hydrophobicity and roughness recovery, three samples of Lotus corniculatus were subjected to 1, 3, or 5 "Ice-covering–deicing" cycles, respectively.

For this study, minor physical damage, severe physical damage, chemical damage, and "over-icing and deicing" damage applied 1, 3, or 5 times were numbered as groups A-F.

## Results and discussions

### Wettability and surface morphology

To study the hydrophobicity recovery of lotus leaves after damage, the wettability and surface roughness of lotus leaves before damage were measured and used as benchmarks to study the hydrophobicity recovery degree.

**Fig. 6:**
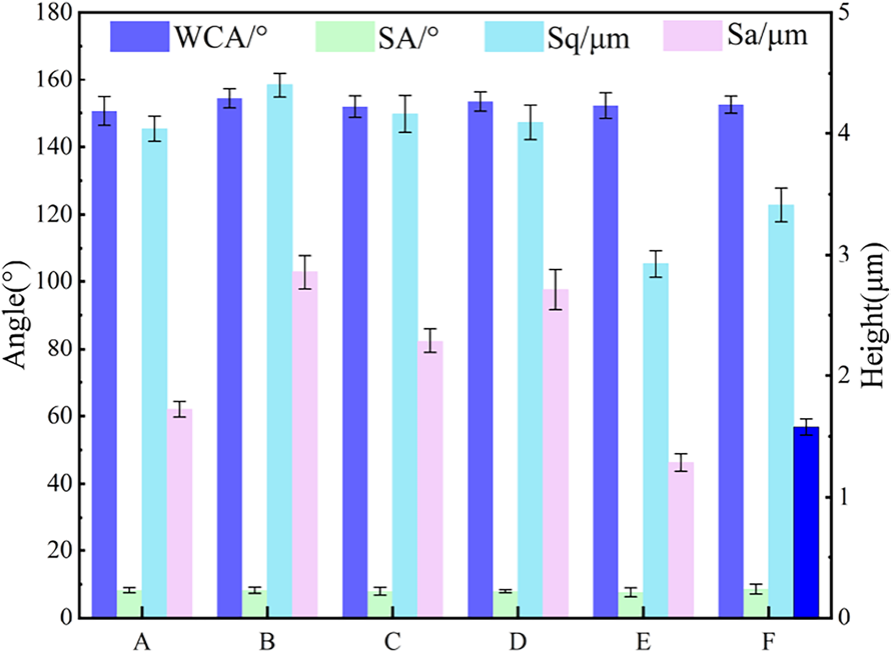
Initial test results of the control sample

The measurement results are shown in Fig. 6, which shows that the average WCA of the lotus leaf surface in Groups A–F was 151.78° and the average rolling angle (SA) was 7.3°, confirming that the undamaged lotus leaf surface was superhydrophobic and had good self-cleaning properties. The Sa of the lotus leaf samples ranged from 3.85 to 4.35 μm, and the Sq ranged from 4.37 to 4.82 μm, with some variations but generally consistent with biodispersibility.

### Wettability and surface morphology after damage

Immediately after the damage was applied, the lotus leaf surface was tested to obtain the surface wettability and roughness parameters after applying different damage types.

(1) *Wettability*

**Fig. 7:**
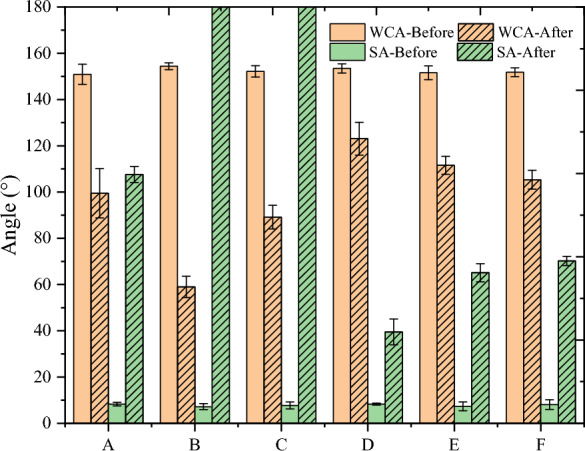
The wettability before and after damage

Wettability measurements are shown in Fig. 7. The orange and green colors represent the WCA and roll angle, respectively; the unshaded bar graphs are the pre-damage measurements, and the shaded bar graphs indicate the post-damage measurements.

**Fig. 8:**
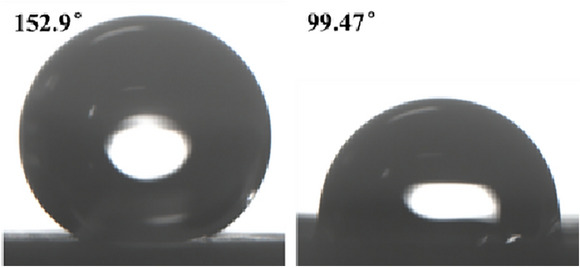
Water contact angle before and after minor physical damage

The results of the wettability test for the Group A samples (minor physical damage) are shown in Fig. 8. The WCA decreased by 33.08% from 152.9° to 99.47°, indicating a loss of superhydrophobicity. The SA increased significantly from 7.4° to 107.6°. Roll angles greater than 90° meant that deionized water could not be dislodged from the surface even when the surface was placed vertically, indicating a complete loss of self-cleaning ability.

**Fig. 9:**
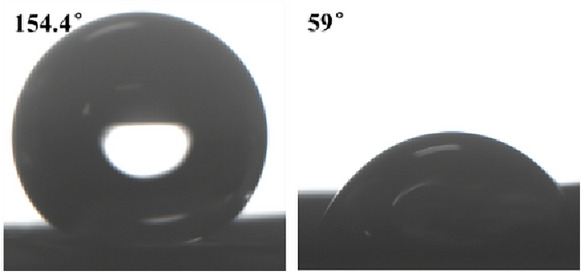
Water contact angle before and after severe physical injury

The results of the wettability test for Group B samples (severe physical damage) are shown in Fig. 9. The WCA decreased from 154.4° to 59.0°, losing its hydrophobicity; the decrease was much greater than that of the minor physical damage. The SA increased from 7.2° to more than 180°; even if the surface was turned over, the deionized water could not be dislodged, completing the loss of self-cleaning. The results show that the effect of sandpaper abrasion on the wettability of the lotus leaves is much higher than that of the two-component adhesive stripping method.

**Fig. 10:**
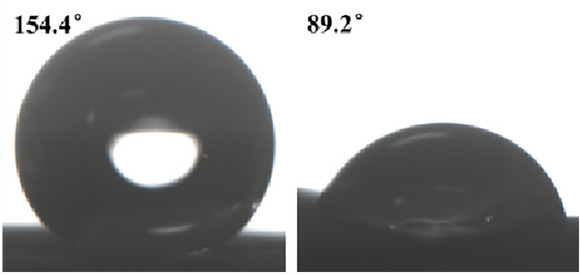
Water contact angle before and after chemical injury

The results of the wettability test for Group C samples (chemical damage) are shown in Fig. 10. The WCA decreased from 152.2° to 89.2°, which indicates that the water-repellent property was lost. The SA increased from 7.7° to more than 180°. Analyzing the effect on the wettability of the lotus leaves, the chemical damage was between minor and severe physical damage. The loss of the lotus leaf's low-energy surface was caused by chloroform dissolution of the micro/nanostructure wax surface. As a result, the effect of chemical damage on wettability is higher than that of minor physical damage. However, severe physical damage not only caused the surface waxes to become damaged but also squeezed the epidermal cells, which contributed to a further decrease in roughness; therefore, the wettability performance was even worse. To verify the accuracy of the above conjecture, it will be analyzed in the next section (surface roughness parameters).

**Fig. 11:**
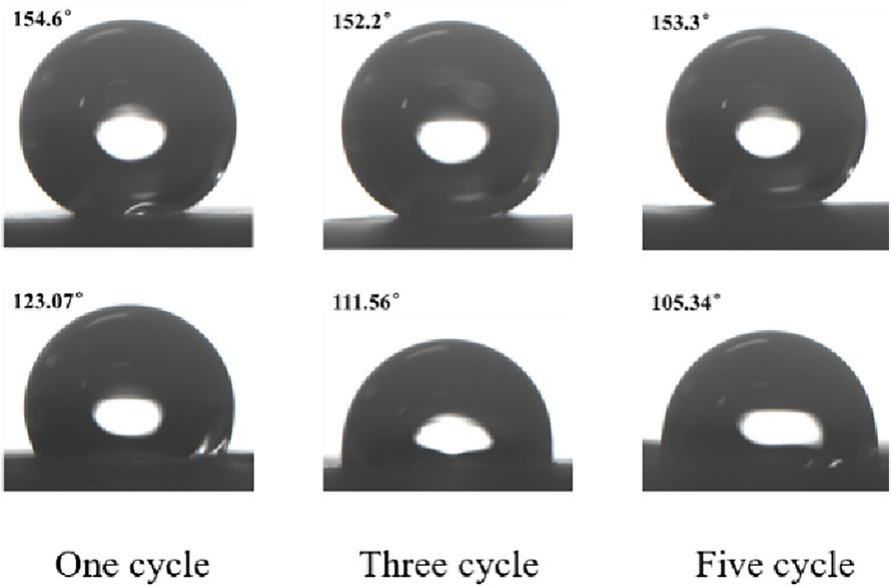
Water contact angle before and after " Ice-covering-deicing " injury

The "ice-covering–deicing" cycle is an integrated process that involves physical and chemical damage. The changes in the WCA after different numbers of "over-icing and deicing" cycles are shown in Fig. 11: after the first "ice-covering–deicing" cycle, the WCA decreases to 123.07°, which indicates that the surface loses its superhydrophobicity but retains a certain hydrophobicity, and the SA increases to 39.5°. After three cycles, the WCA dropped to 111.56° and the SA rose to 65.1°. After five cycles, the WCA dropped to 105.34°, and the SA rose to 70.2°. Therefore, the greater the number of "over-icing–deicing" cycles, the greater the change in WCA and SA, and the more severe the decrease in wettability.

**Fig. 12:**
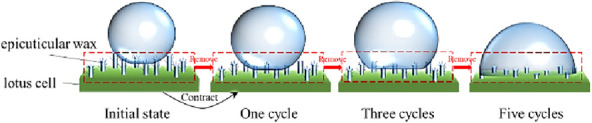
The influence of the icing-deicing cycle

As the number of ice-covering cycles increases, the decrease in wettability slows. This article hypothesizes that the ice cover triggers an "anchoring" effect on the surface micro/nanostructures, which causes the lotus leaf cells to shrink as they are squeezed. The wax layer fixed to the epidermis is destroyed during the deicing process. Under the combined effect of these two factors, the surface roughness decreases, leading to a decrease in wettability. Figure 12 shows the effect of the number of "ice-covering–deicing" cycles on the lotus leaf surface.

(2) *Surface roughness parameters*

**Fig. 13:**
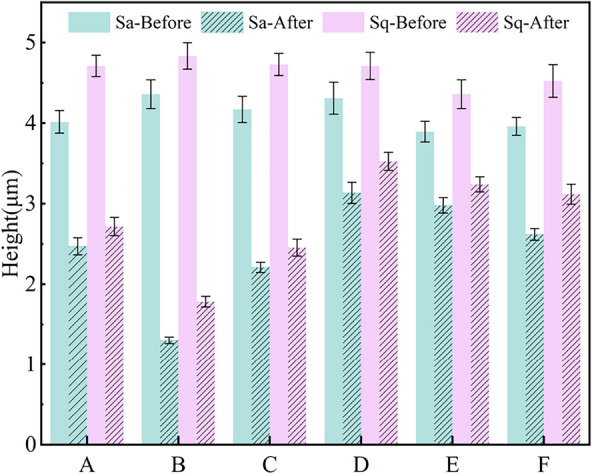
Roughness parameters before and after damage

Wettability measurements revealed several phenomena. To investigate the reasons for these phenomena, the surface roughness parameters were analyzed. The results of roughness parameter measurements before and after damage are shown in Fig. 13.

**Fig. 14:**
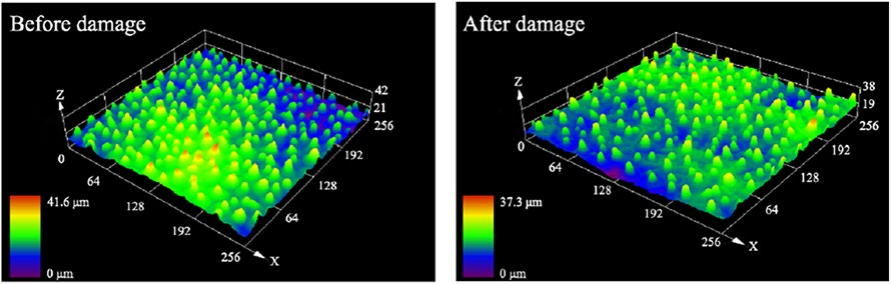
Confocal micrograph before and after minor physical damage

After minor physical damage to Group A, the Sa decreased from 4.018 to 2.434 μm, a decrease of 39.42%. The Sq decreased from 4.709 to 2.711 μm, a decrease of 42.43%. Confocal microscopy images of the samples in Group A are shown in Fig. 14.

**Fig. 15:**
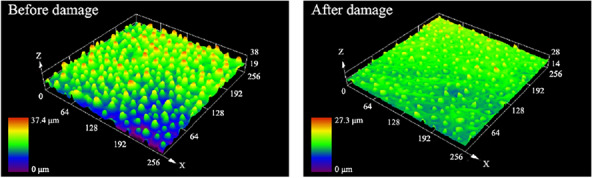
Confocal micrograph before and after severe physical damage

Figure 15 shows the confocal microscopic images of severe physical damage for the samples in Group B. Compared with the minor physical damage samples, the decreases in Sa and Sq on the lotus leaf surface are more significant. The Sa decreases from 4.357 to 1.279 μm, while the Sq decreases to 1.762 μm.

The confocal micrographs revealed that the height of the protrusions on the lotus leaf surface after severe physical damage decreased dramatically and became flat overall, which is consistent with the test results of the surface roughness parameter. This result also indicates that severe physical damage has a greater effect on the surface roughness, which is consistent with the results of the wettability test.

**Fig. 16:**
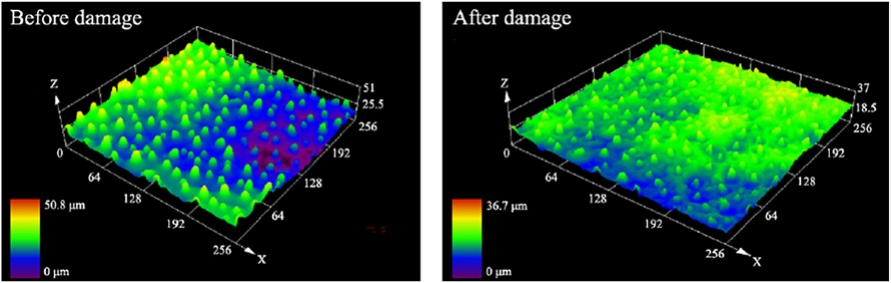
Confocal micrograph before and after severe chemical damage

After chemical damage to Group C samples, the Sa decreased from 4.183 to 2.193 μm; the Sq decreased to 2.451 μm. In terms of the effect on surface roughness, the chemical damage was in between severe and minor physical damage. To explore the reason, the confocal images of Group C samples before and after applying damage were analyzed, as shown in Fig. 16.

As can be seen in Fig. 16, the surface becomes flat after applying chemical damage. Although the absolute peak values of the protrusions appear to be lower than the images of severe physical damage, the values of the surface roughness parameters Sa and Sq are determined by the point-to-center surface distance within the contoured surface. Due to the overall lower height of the image shown in Fig. 16, resulting in a higher relative protrusion height and a greater surface roughness parameter, the wettability is better than that of the lotus leaf after severe physical damage. Again, this matches the results of the surface wettability tests.

**Fig. 17:**
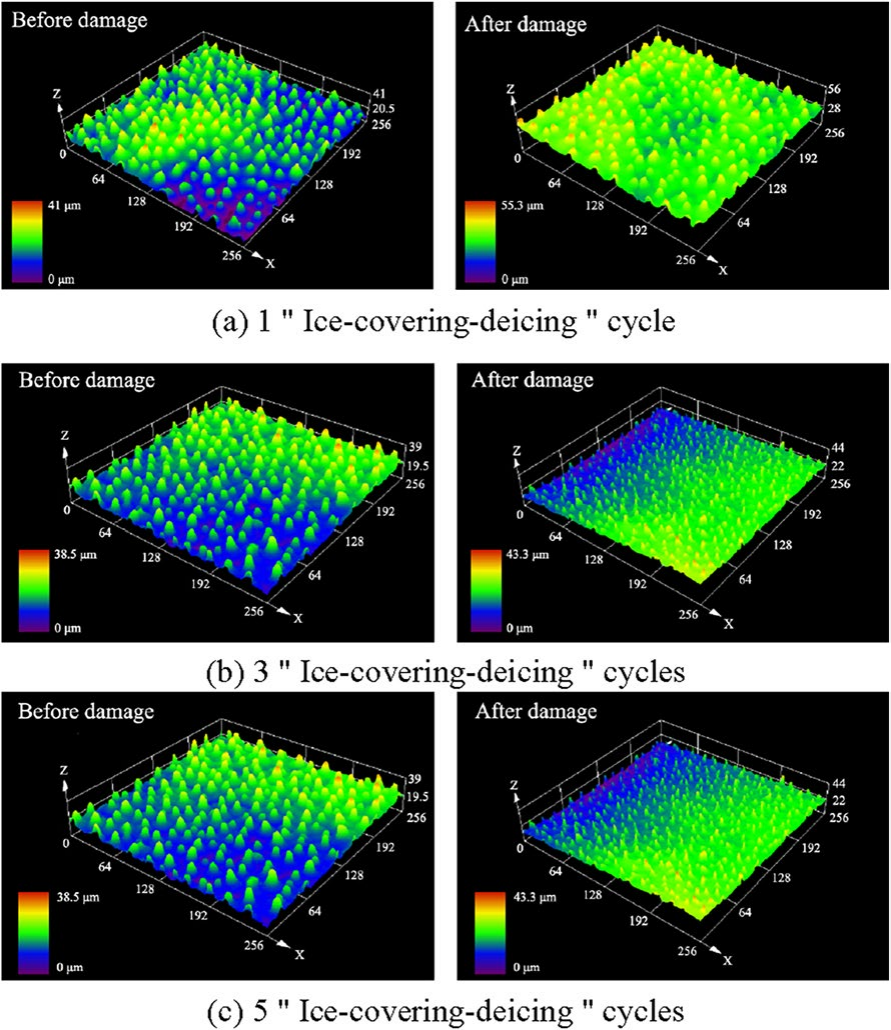
Confocal images of the surface of the lotus leaf after different numbers of " Ice-covering-deicing " cycles

The confocal images of the surface of the lotus leaf after different numbers of "ice-covering-de-icing" cycles are shown in Fig. 17. The changes in the surface morphology of the lotus leaf after 1, 3, or 5 cycles of "ice-covering–deicing" are shown in Fig. 17a, b, c. The Sa decreased to 3.123 μm, 2.682 μm, and 2.624 μm, with decreases of 27.56%, 30.91%, and 33.99%, respectively. The Sq decreased to 3.542 μm, 3.221 μm, and 3.113 μm, with decreases of 25.1%, 26.36%, and 31.10%, respectively. The absolute values of Sa and Sq decrease with an increase in the cycle number. At the same time, the greater the number of "ice-covering–deicing" cycles, the more severe the decrease in Sa and Sq.

### Processes of recovery of water repellency and surface roughness

The first test after the damage was applied was recorded as 0 h. Times of 0 h, 2 h, 4 h, 8 h, and 24 h after the damage was applied were selected as the test times. Tests were conducted every 24 h thereafter until the lotus leaf surface returned to a hydrophobic state or the test results stabilized.

(1) *Minor physical damage*

**Fig. 18:**
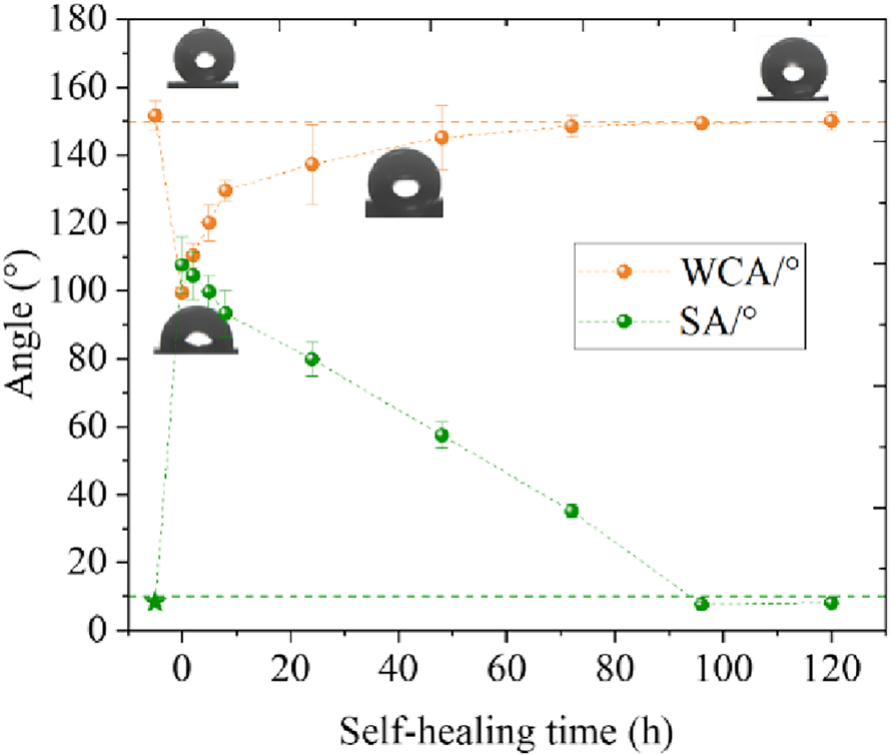
Recovery process of surface hydrophobicity with time after minor physical damage

Measurements of surface hydrophobicity were performed at the times described above after the application of minor physical damage, as shown in Fig. 18. The first result from the left is the result of the test before 0 h and represents the initial value for each sample.

As can be seen in Fig. 18, the WCA recovery process in the case of minor physical damage can be divided into two parts. In the first part, a period of rapid recovery occurs within 8 h after the damage is applied. In this phase, the WCA of the lotus leaf surface recovered from 54.92 to 85.88% of the initial value after minor physical damage. The second part was from 8 h until the end of the measurement. During this period, the recovery rate slowed significantly. With an increase in time, the recovery speed gradually decreased, and the curve flattened. The recovery curve showed a clear exponential function characteristic of a gradual decrease in recovery speed with time. The WCA recovered to more than 150° 72 h after minor physical damage.

The SA did not have a rapid recovery phase similar to the WCA, but the recovery rate was relatively stable. It returned to below 10° after 96 h. At this point, the hydrophobicity and self-cleaning properties of the lotus leaf were fully restored. Thereafter, the SA of the lotus leaf surface recovered slowly.

**Fig. 19:**
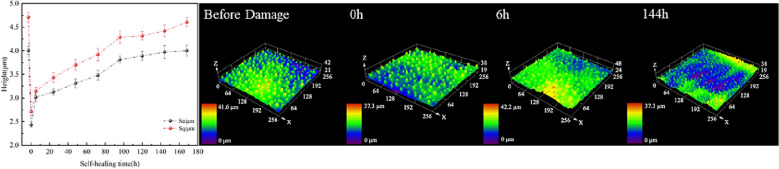
Recovery process of surface roughness parameters after minor physical damage and typical confocal image

To better understand the surface morphology recovery characteristics of lotus leaves after different injuries, four important time points were selected: initial state (before injury), injury completion (0 h), short recovery (6 h), and steady state. The roughness recovery process was captured through confocal microscope images. The surface roughness recovery process for minor physical damage is shown in Fig. 19. The recovery process of the two roughness parameters is the same. In the period from 0–8 h, there also exists an obvious rapid recovery phase for the roughness parameters, and then the recovery process gradually slows. This is consistent with the hydrophobicity recovery trend.

(2) *Severe physical damage*

**Fig. 20:**
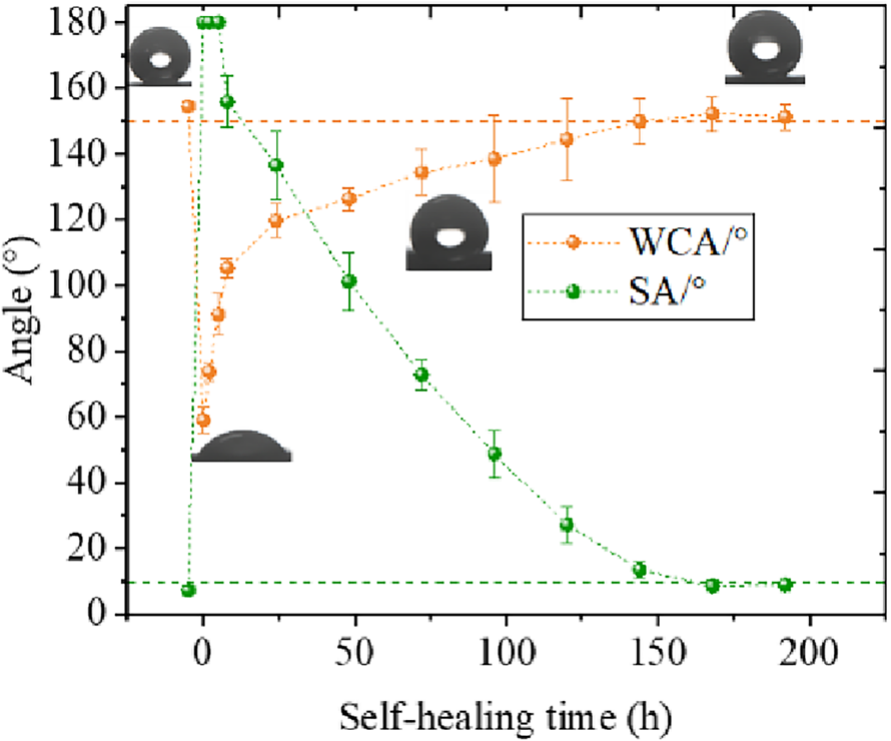
Recovery process of surface hydrophobicity with time after severe physical damage

The contact angle recovery process for severe physical damage is shown in Fig. 20. The WCA recovery process is similar to that of minor physical damage. There are also two distinct phases. During the initial recovery phase after injury, the WCA values recovered dramatically. The WCA recovered from 59.0° to 105.3° in 8 h, recovering to 68.20% of the initial value. Because the damage was more severe than minor damage, the hydrophobicity loss degree was greater, resulting in a longer required recovery time. At the 144th hour of testing, the WCA recovered to 149.8°.

One particular phenomenon was that the roll angle measurements were all greater than 180° within 6 h of injury. This means that the deionized water droplets cannot roll off even if the sample is turned over. Measured at 8 h post-injury, the SA decreased to 155.8°. As time increased, The SA continued to decrease and gradually stabilized. At the 168 h measurement, the SA dropped below 10°. When the SA recovered to below 10°, the WCA recovery rate decreased. Thereafter, the SA value hardly changed.

**Fig. 21:**
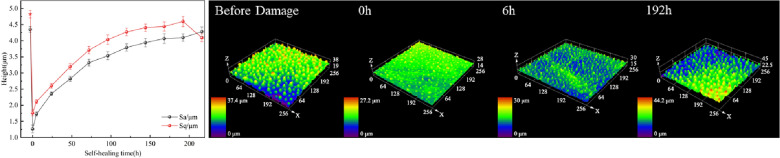
Recovery process of surface roughness parameters with time after severe physical damage and confocal image

Figure 21 shows the recovery of lotus leaf roughness in the case of severe physical damage. The recovery of the roughness parameter also shows a gradual slowdown, which is consistent with the hydrophobicity recovery curve. The fluctuations in roughness parameters are within reasonable limits, considering the decentralized nature of this biological system. The large decrease in the measurement of Sq at 216 h is due to a difference in the chosen sampling location.

(3)* Chemical damage*

**Fig. 22:**
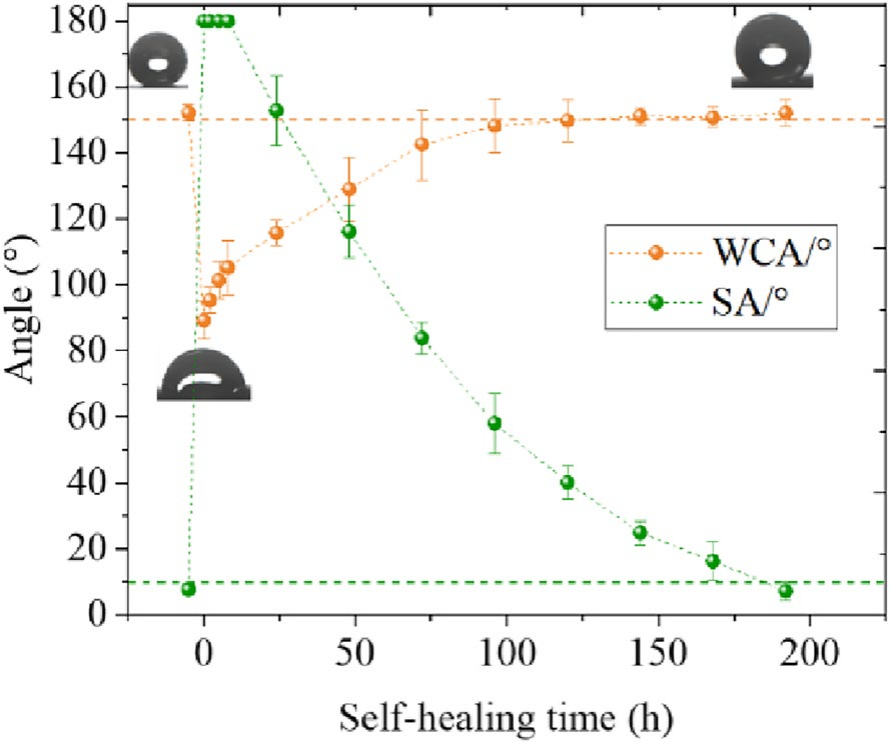
Recovery process of surface hydrophobicity with time after chemical damage

Figure 22 represents the variation curve of the hydrophobicity parameter in the case of chemical damage. The WCA recovery process shows typical exponential characteristics. The recovery speed decreases gradually with an increase in the recovery degree. This feature is consistent with the theory of surface wax diffusion. During the 0–8 h recovery phase, unlike the rapid recovery phase that exists for physical injuries, the recovery curve for chemical injuries is smoother. This is a very interesting phenomenon. This means that chemically damaged WCA has a lower recovery rate than physical damage during the initial recovery phase. The recovery rate was also slower than for physical injury. The WCA, after chemical injury, only recovered from 89.2° to 105.18° within the first 8 h and eventually recovered to about 150° within 120 h.

After the chemical injury, the SA remained above 180° in all 0–8 h recovery phases until the 24th hour, when it dropped to 152.9°. Thereafter, the SA started to recover slowly, and the recovery process conformed to the characteristics of an exponential function. It finally recovered to 7.2° after 192 h and regained its self-cleaning properties.

**Fig. 23:**
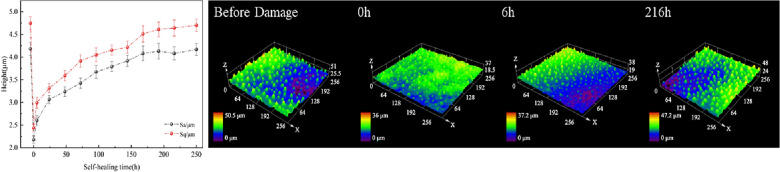
Recovery process of surface roughness parameters with time after chemical damage and typical confocal images

Figure 23 represents the recovery process of roughness parameters with time in the case of chemical damage. The recovery process is similar to the physical damage process.

(4) *"Ice-covering-deicing" cycle damage*

**Fig. 24:**
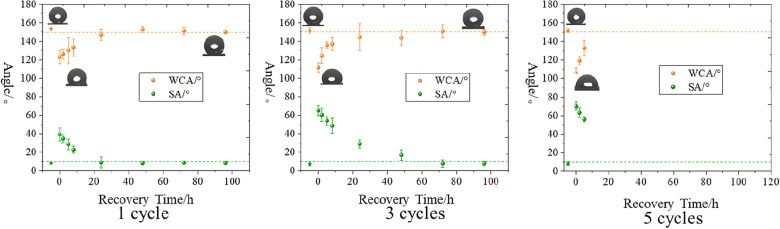
Recovery of hydrophobicity after 1, 3, and 5 cycles of "Ice-covering-deicing"

To better guide the preparation of hydrophobic materials with fast recovery and self-growth properties, the hydrophobicity and roughness recovery process after different "ice-covering and deicing" cycle damage was especially studied. The recovery hydrophobicity process after different cycle numbers of "ice-covering and deicing" is shown in Fig. 24.

The WCA of the lotus leaf was fully restored to more than 150° after 48 h for the lotus leaf plants damaged by one "ice-covering–deicing" cycle. After 24 h, The SA decreased to below 10°. At this point, the self-cleaning properties of the leaf surface were fully restored. In the case of the first "ice-covering–deicing" cycle, the recovery of SA was completed before the WCA.

After three cycles of "over-icing and deicing" (about 72 h), the surface WCA was completely restored to its initial value, and the SA was also restored to its initial value. There was a period of rapid recovery in the WCA in the case of three `ice-covering–deicing' damage cycles. The lotus leaf surface was terminated after five icing–deicing damage cycles. Even when part of the surface regained its hydrophobicity, it gradually wilted. The reason was that the plant died as a result of the low temperature and was no longer able to provide energy and synthesize the waxes necessary for surface morphology recovery.

**Fig. 25:**
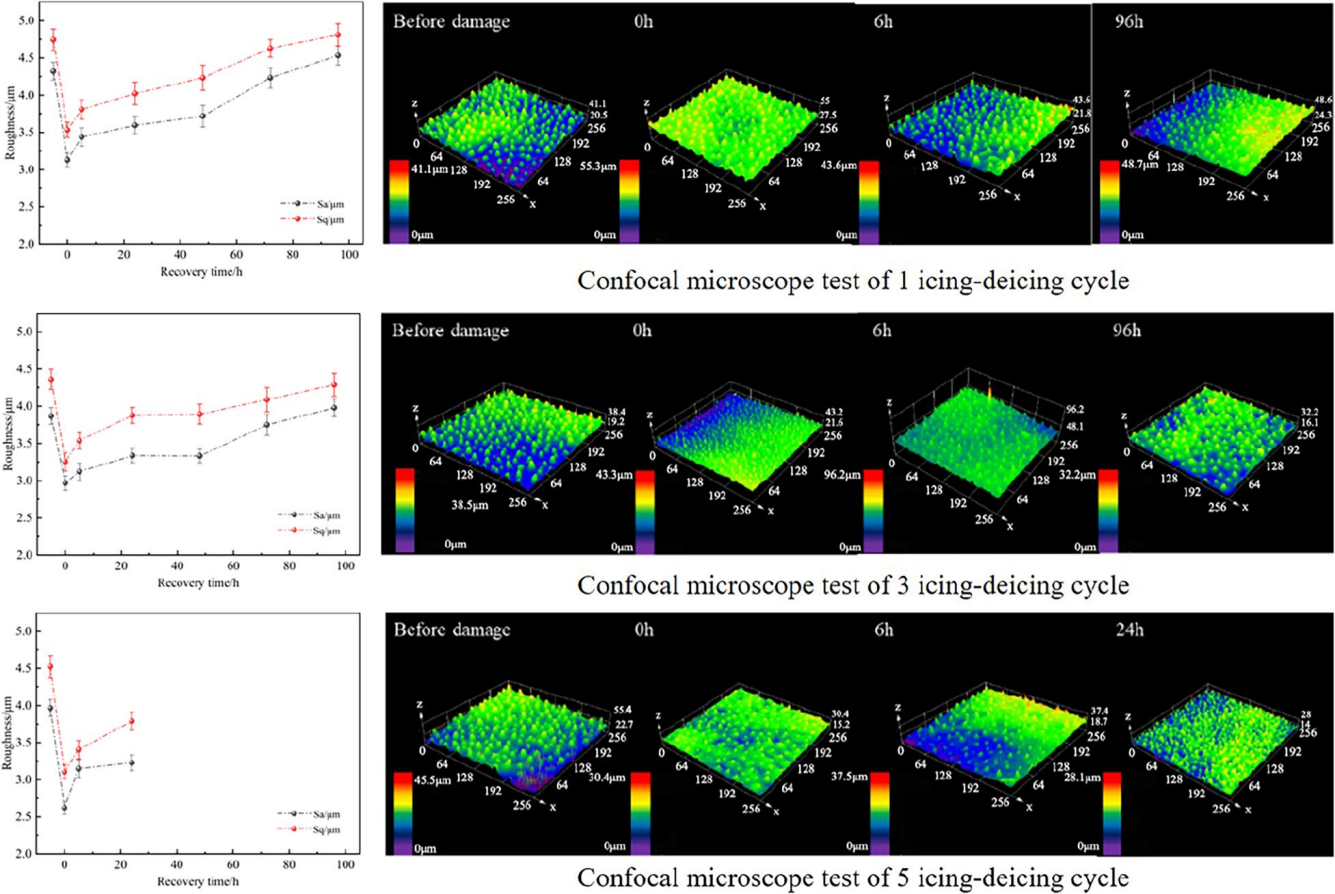
Surface roughness recovery process after 1, 3, and 5 cycles of icing and deicing and typical confocal image

Figure 25 shows the roughness recovery process after different numbers of "ice-covering–deicing" cycles. Based on the confocal image before damage application, it can be seen that the columnar protrusions on the surface are relatively round in the initial state. Observation of the images at 0 h revealed that the height of the columnar protrusions decreased dramatically and became more abrupt and steeper after "ice-covering–deicing." This is despite the fact that the three samples have slightly different initial values due to the fact that they are biological systems. However, the higher the number of "ice-covering–deicing" cycles, the smaller the results of the roughness parameters. As the recovery time increased, the morphology of the surface protrusions gradually became rounded again.

It is important to note that the lotus leaf dies after five "ice-covering–deicing" cycles and no longer retains the ability to produce wax. Therefore, the image 24 h after recovery was chosen as the steady state moment. In the initial state, the columnar protrusions on the surface are relatively rounded. After damage application, the height of the columnar protrusions decreases sharply and becomes more abrupt.

## Analysis of self-recovery mechanism after damage to lotus leaf surface

### Growth process of lotus leaves after surface damage

**Fig. 26:**
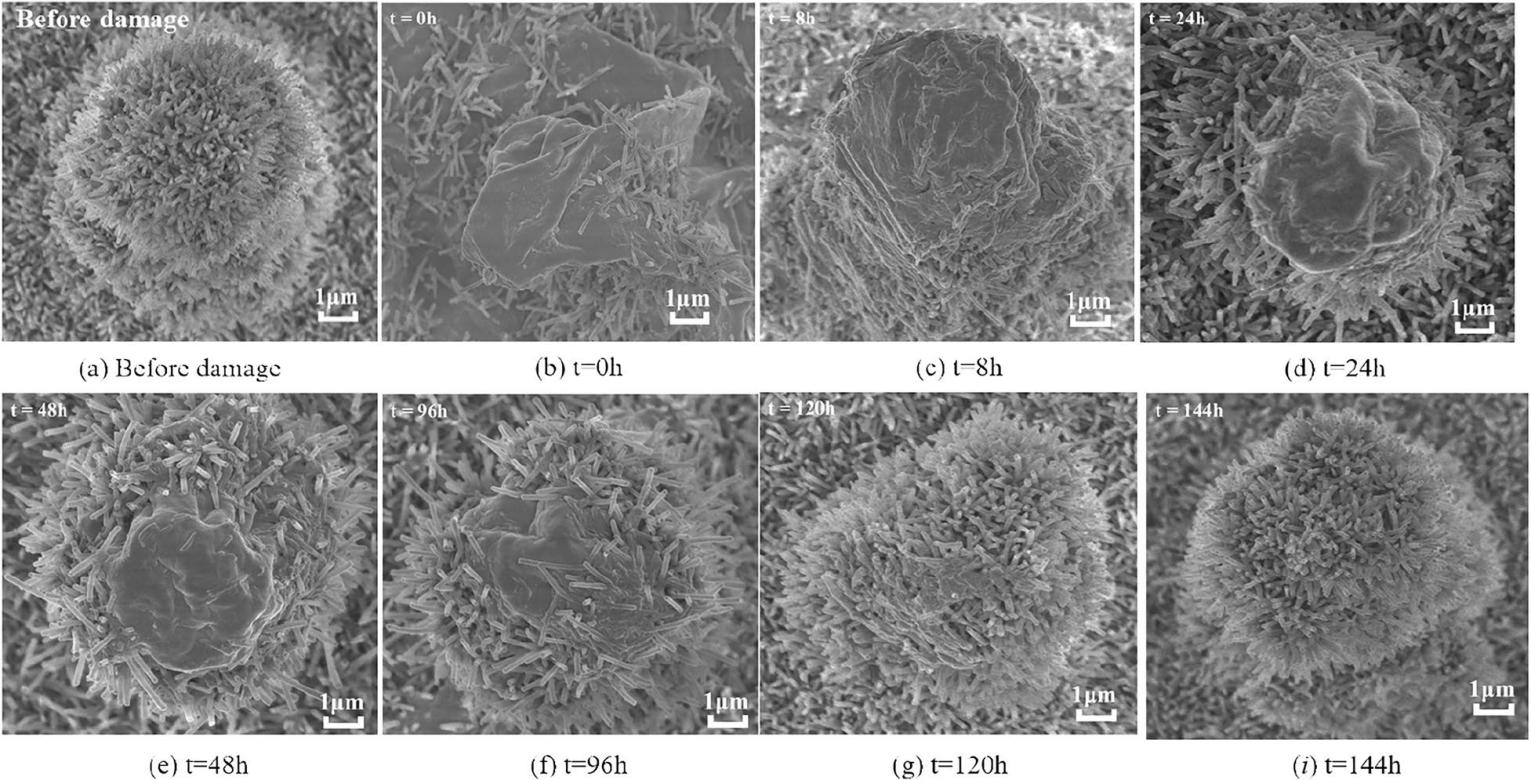
Surface morphology with different recovery times after severe physical damage

To gain a deeper understanding of the wax growth process on the lotus leaf surfaces, the natural growth, as well as the restoration process of lotus leaves after severe physical damage, was observed. During the restoration process, the lotus leaf surface was cut off in intervals. After drying, the lotus leaf surface morphology was measured using a projected scanning electron microscope. The results of the surface topography tests at different recovery times are shown in Fig. 26.

**Fig. 27:**
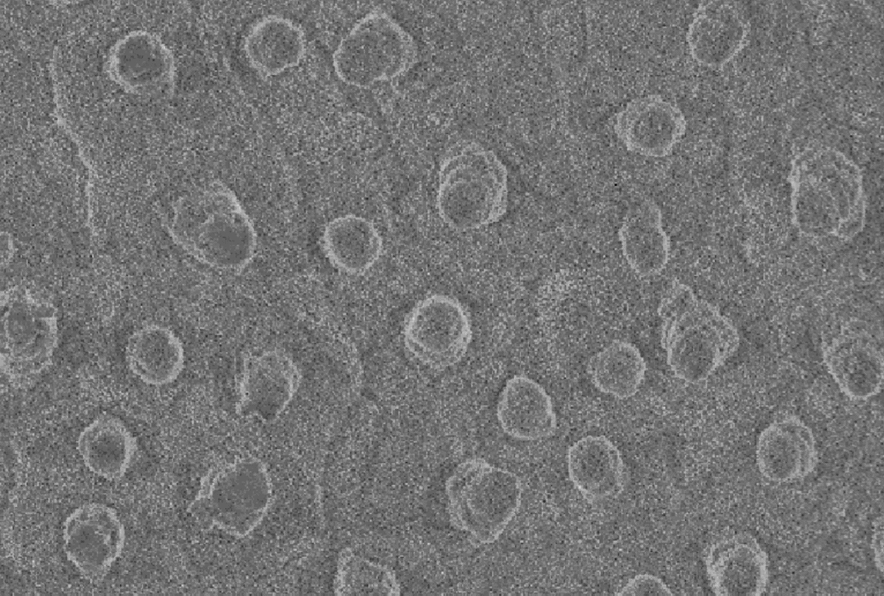
Surface roughness structure of lotus leaves

Before damage application, the micrometer protrusions on the lotus leaf surface and the wax tubules attached to the protrusions generally formed a rounded surface structure. Measurements were taken immediately after damage application, as shown in Fig. 26b. The recovery of the micrometer protrusions at different time periods is shown in Fig. 27*c*~*i. *The wax on the lotus leaf surface was disrupted, and the wax tubules on and around the protrusions were drastically reduced. The micrometer-scale structure supporting the wax tubules was also deformed and no longer remained columnar. However, the micrometer-scale structure did not disappear, indicating that the composition of the micrometer-scale structure should be different from that of the wax tubules.

Then, the surface micrometer protrusions began to recover gradually, and the wax began to grow. After 8 h of recovery, the surface micrometer protrusions returned to a normal state. The lotus leaf surface around the protrusions was re-covered by wax tubules, which began to grow upward gradually. With an increase in recovery time, the micrometer protrusions on the lotus leaf surface were gradually covered by wax tubules, forming a micro/nanostructure comprising multiple layers.

### Definition of two-scale roughness

To analyze the self-growth mechanism of lotus leaf surface after damage, different roughness scales were defined based on the test results. The damage types are classified according to the surface damage mechanism and the hydrophobicity recovery and self-growth mechanism of the lotus leaf surface after different damage types are applied. Finally, the key factors affecting the WCA and SA are analyzed.

After applying chemical damage, the CA becomes larger due to the solubilizing effect of chloroform on the wax surface layer, leading to a sharp increase in also surface energy [21]. If the surface roughness of the lotus leaf is comprised entirely of wax structures, the surface roughness should decrease to nearly 0 after chemical damage dissolves the wax structures. However, the test results show that the lotus leaf surface maintains a certain degree of roughness after chemical damage application, which is even higher than that of the roughness test results after severe physical damage. This phenomenon suggests that the roughness of the lotus leaf is not only provided by micrometer protrusions and wax nanostructures.

Analyzing the microscopic images on the lotus leaf surface, obvious micron-sized protrusions can be seen. However, the micron-sized protrusions that exist on top of the structures (grooves), which have their roughness, as shown in Fig. 27.

This part is not a rough structure composed of plant epidermal waxes, is not subject to chloroform immersion, and is derived from plant cells [17]. It is generally recognized that hydrophobic surfaces possess micro/nanostructures. In contrast, the results of this study demonstrate that micron protrusions are not the only source of micro/nanostructures. The self-contained roughness of plant epidermal tissues is also an important source of micron-scale protrusions.

Therefore, micron protrusions composed of plant cells and micron-sized epidermal waxes can be defined as micro-roughness, while nanostructures attached to micron protrusions are defined as nano-roughness [22].

### Main factors affecting hydrophobicity

To analyze the recovery process of static contact angle (WCA) and rolling angle (SA) following various types of damage, we delineated three key stages: damage completion (0 h), initial recovery (0–8 h), and stabilization. Following damage completion, the surface enters a fully wetted state, known as the Wenzel state. During this phase, WCA decreases rapidly while SA swiftly increases to nearly 180°. Subsequently, during the initial recovery period (0–8 h), the surface transitions into a mixed state. Although WCA begins to increase, SA undergoes a slow evolution. Upon reaching a stable state, the surface achieves superhydrophobicity (Cassie-Baxter state), with SA reverting to its pre-damage level. Notably, the restoration of the rolling angle on the lotus leaf surface occurs no earlier than the recovery time of the static contact angle. Furthermore, the process of rolling angle recovery does not exhibit a rapid restoration phase, indicating that the cells responsible for the pronounced recovery of the static contact angle undergo gradual re-expansion, thereby minimally impacting the roll angle recovery process.

Combining the recovery characteristics of the WCA and SA and the two-scale roughness definition, the main factors affecting the WCA and SA can be characterized as different scales of roughness.

The microscopic roughness of the lotus leaf surface is composed of epidermal cells and micron-sized epidermal wax crystals. After severe damage, the lotus leaf first re-expands crushed epidermal cells and regenerates epidermal wax crystals to form micrometer-scale surface structures. As the epidermal cell re-expansion process ends, the epidermal waxes continue to regenerate to form nanoscale surface structures on micrometer-sized protrusion surfaces. The microstructure recovery is usually faster than that of the nanostructure, and the recovery of the WCA is also faster than that of the SA. Therefore, the WCA is mainly determined by microstructure roughness, while the nanostructure roughness mainly determines the SA recovery process.

### Analysis of growth mechanisms

**Fig. 28:**
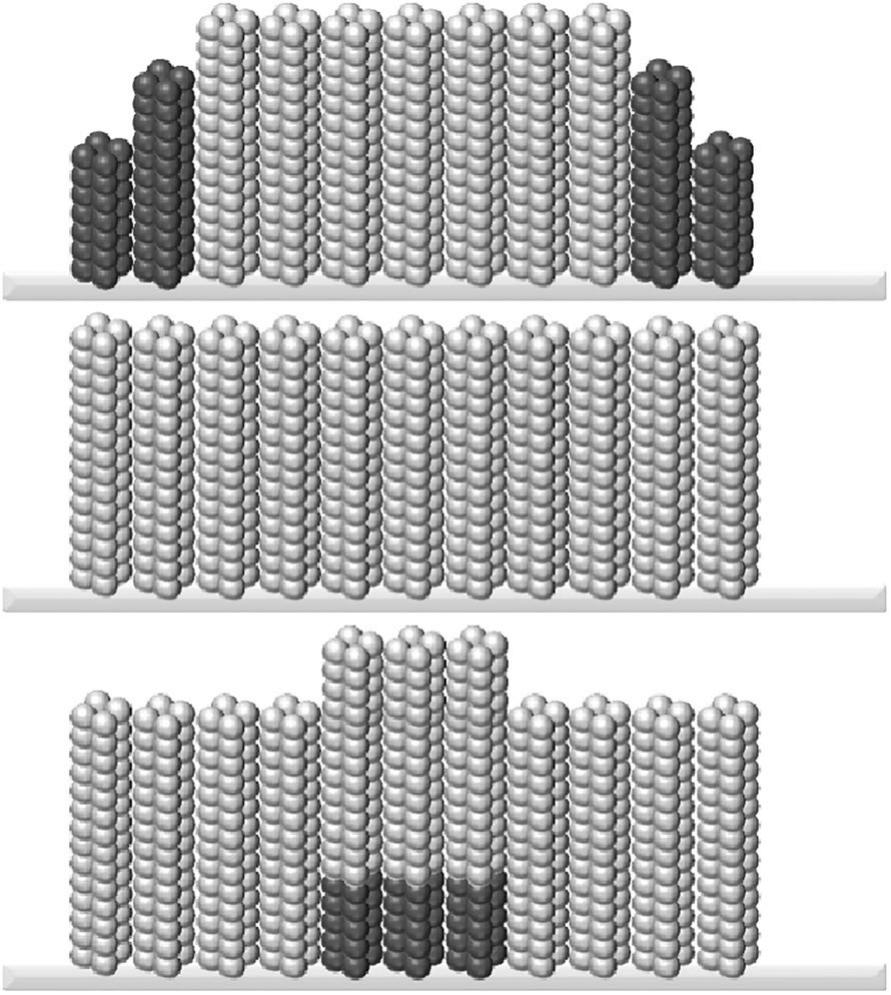
Self-assembly process of waxy molecules

Based on the test results, although the absolute value of the decrease in the surface roughness parameters resulting from different damage types and different numbers of "ice-covering–deicing" cycles differ, the recovery of the surface roughness, in general, shows an exponential function with time, characterized by a "first fast and then slow" recovery process. This characterization is consistent with typical solid diffusion models. Whether only the surface wax layer was damaged or the surface wax layer was damaged with simultaneous epidermal cell extrusion, epidermal waxes on the lotus leaf surface were removed by the damage. Consequently, the gradient in phytowax concentration on the surface and inside leads to an increase in the transpiration rate at these sites, resulting in the regeneration and replenishment of epidermal waxes. As epidermal waxes were regenerated and replenished, the transpiration rate slowed as the gradient between internal and external phytowax concentrations decreased. The hydrophobicity recovery rate slowed with increasing time. The results of this study showed that most plants were able to fill the cavities in the epidermal wax layer [23]. The formation process for the unique wax layer structure is also formed by the self-assembly of wax molecules during diffusion. New wax molecules are preferentially added to the edges of the existing layer to form a lamellar structure. Subsequently, the existing wax layer is elevated by the new wax layer underneath to form different roughnesses, as shown in Fig. 28. The same wax crystal micromorphology forms when the wax composition is almost the same as when the wax layer initially formed [24].

## Conclusion

In this study, we investigated alterations in water repellency and surface roughness on lotus leaf surfaces following various damage types, examining the corresponding recovery processes over time. We aimed to analyze the restoration dynamics after different forms of damage and to unveil the mechanisms underlying the recovery of water repellency on lotus leaf surfaces post-ice-covering damage. The key conclusions drawn from our investigation are as follows:

1. The nuanced roughness exhibited on the lotus leaf surface stems from the interplay of epidermal cells and micrometer-scale epidermal wax crystals. The compromised integrity of a lotus leaf initiates a sequence wherein extruded epidermal cells undergo re-expansion, if present, and concurrently, the regeneration of epidermal wax crystals takes place, constructing a micrometer-scale surface configuration. Upon completion of the epidermal cell re-expansion phase, the regeneration of epidermal wax persists, contributing to the formation of a nanometer-scale surface structure. Generally, the recovery of the microstructure tends to outpace that of the nanostructure. The WCA exhibits a notably higher correlation with the microstructure, while the nanostructure predominantly influences the determination of the SA.
2. The restoration of surface hydrophobicity in lotus leaves is contingent upon two key factors: the re-expansion of epidermal cells and the recovery of the epidermal wax layer. Depending on the nature of the damage, the recovery process can be delineated into two distinct types: (a) Removal of the surface wax layer exclusively. In such instances, the WCA recovery of the lotus leaf exhibits characteristic exponential behavior, with no abrupt changes during the initial stages. (b) Removal of the surface wax layer concurrent with epidermal cell extrusion. In this scenario, the hydrophobicity recovery unfolds in two discernible phases. The initial phase involves the re-expansion of cells and the regeneration of micrometer-sized wax structures, characterized by a rapid and pronounced recovery process. Subsequently, the epidermal waxes persist in regenerating on the micrometer-sized structures, forming nanometer-sized surface structures.

Based on the insights gained from the recovery process of the lotus leaf surface, the design concepts for a self-growing hydrophobic surface can be outlined as follows:

1. Leveraging Viscoelastic Properties of Shape Memory Polymer: Utilize the viscoelastic effect inherent in shape memory polymers to retain and recall micrometer-scale rough structures. Construct a surface featuring micrometer-scale rough structures using the shape memory polymer as a base material, facilitating the autonomous regeneration of the hydrophobic surface at the micrometer scale.
2. Incorporating Low-Surface-Energy Substances in the Matrix: Integrate low-surface-energy substances within the material matrix. Exploit the migratory properties of these substances to facilitate the formation of nanometer-scale rough structures coupled with low-surface-energy characteristics. Subsequently, hydrophobicity recovery is achieved through a diffusion-driven process following any inflicted damage.

These design ideas draw inspiration from the natural recovery mechanisms observed in lotus leaves, providing innovative approaches for creating self-growing hydrophobic surfaces with enhanced resilience and functionality.

## Data Availability

No datasets were generated or analysed during the current study.
